# Intensivist-led on-call service: key step in mortality reduction

**DOI:** 10.1186/cc9894

**Published:** 2011-03-11

**Authors:** NJ Harris, H Kilner, A Krishnamurthy, P Bishop

**Affiliations:** 1 The Princess Alexandra Hospital NHS Trust, Harlow, UK

## Introduction

We conducted an audit to determine whether a change to a dedicated intensivist rota in our district general hospital ICU improved patient outcome. Our unit, like many others around the country, had historically been covered out of hours by anaesthetists rather than specialists in intensive care medicine. This audit therefore had potentially far-reaching implications for many other similar ICUs in the UK.

## Methods

We conducted a retrospective analysis on data obtained from the ICNARC database, patient notes, drug charts and ICU charts over two cycles. The first ran from 1 December 2008 to 31 January 2009, when the conventional on-call consultant rota was still in operation. The second ran from 1 January 2010 to 31 March 2010, following implementation of a dedicated intensivist rota. Our primary outcome measure was unit mortality. We analysed a further eight parameters as indirect markers of good clinical practice. These were tidal volume, urine output, glycaemic control, lactate, mixed venous oxygen, and appropriate prescription of gastric protection, antibiotics and venous thromboembolism prophylaxis.

## Results

Patient demographics were similar between the two cohorts under investigation, but the mean admission APACHE II score was found to be significantly lower following the rota change, as shown in Table 1. This reduced inpatient unit mortality from 39% in cycle 1 to 25% in cycle 2. However, the change to an intensivist rota made little difference to our markers of good clinical practice.

**Table 1:** Patient demographics

Factor	Cycle 1	Cycle 2
Subjects	82	76
‰ male	48	59
Mean age (years)	63	64
Age range (years)	19 to 91	21 to 91
APACHE	24.8	17.6
Range	5 to 36	5 to 42

## Conclusions

Our study suggests that the improvement to unit mortality was secondary to patient selection, rather than a fundamental change in clinical practice within the ICU. This indicates that a dedicated rota, in which consultant intensivists lead on out-of-hours referrals, reduces the number of inappropriate admissions to the ICU.

